# Generic placement of the Neotropical species of “ *Phragmatobia*” (Erebidae, Arctiinae), with a remarkable matrivorous species from the Peruvian Andes

**DOI:** 10.3897/zookeys.149.2382

**Published:** 2011-11-24

**Authors:** B. Christian Schmidt, Josef J. De Freina

**Affiliations:** 1 Canadian Food Inspection Agency, Canadian National Collection of Insects, Arachnids and Nematodes, K.W. Neatby Bldg., 960 Carling Ave., Ottawa, ON, Canada K1A 0C6; 2 Eduard-Schmid Str. 10, D-81541, Munich, Germany

**Keywords:** Microptery, flightlessness, matrivory, biased sex ratio, Spilosomini, Spilosomina, Arctiidae, Neotropics, taxonomy, *Lachana*, *Metacrias*, *Phaos*, *Pyrrharctia*, Gondwana, circumantarctic

## Abstract

*Phragmatobia* Stephens is briefly reviewed and a diagnosis is provided. The South American species currently placed in *Phragmatobia* Stephens are revised to two new genera, *Andesobia* Schmidt and De Freina, **gen. n.**, and *Patagobia* Schmidt and De Freina, **gen. n.** (subtribe Spilosomina). Both *Andesobia* and *Patagobia* exhibit adaptations to high altitude habitats, including micropterous females in *Andesobia* (*Patagobia* females are unknown) and diurnal flight of males. The adults, immature stages, and mating behaviour of *Andesobia jelskii* (Oberthür, 1881) are described. Males of *Andesobia jelskii* enter the female cocoon to mate, and the micropterous, flightless females remain in the cocoon following oviposition where newly hatched larvae feed initially on the female’s body.

Four species are included in *Andesobia*, *Andesobia jelskii* **comb. n.** (= *Paracles imitatrix* Rothschild, 1922, **syn. n.**), *Andesobia flavata* (Hampson, 1901), **comb. n**., *Andesobia boliviana* (Gaede, 1923), **comb. n.** (=*Turuptiana flavescens* Rothschild, 1933, **syn. n.**), and *Andesobia sanguinea* (Hampson, 1907), **comb. n.** *Patagobia* includes only *Patagobia thursbyi* (Rothschild, 1910), **comb. n.**, and *Patagobia thursbyi pluto* Toulgoët is relegated to its synonymy. *Patagobia* shows affinities to *Phaos* Walker, 1855 of Australia, *Metacrias* Meyrick, 1886 of New Zealand, and *Pseudophragmatobia* Krüger, 2009 of South Africa, suggesting a common ancestry of circumantarctic origin. *Phragmatobia karsholti* Toulgoët, 1991 is transferred to *Venedictoffia* Toulgoët, **comb. n**., an unrelated genus that is removed from subtribe Arctiina and provisionally placed in the Phaegopterina. *Phragmatobia oberthueri* Rothschild, 1910, described from Tibet, is a synonym of *Lachana alpherakii* (Grum-Grzhimailo, 1891) [Erebidae: Lymantriinae], **syn. n.**, **comb. n.**

## Introduction

Although the Arctiinae are most diverse in the Neotropical realm with 55% of the approximately 11,000 described species globally (Heppner 1991), diversity of the subtribe Spilosomina is relatively low compared to the Oriental and Ethiopian regions. However, large species radiations have occurred in the *Hypercompe* Hübner and *Paracles* Walker generic groups, which together contain about 60% of the Neotropical spilosomine species (Watson and Goodger 1986). Unlike most New World spilosomine genera, which have either predominantly temperate or tropical distributions, the Andean species currently placed in *Phragmatobia* are enigmatic in that they exhibit similarities to the Australian genus *Phaos* Walker, 1855 and Neozealandian genus *Metacrias* Meyrick, 1886(Ferguson 1985). Ferguson (1985) suggested a common Gondwanan ancestry for Andean *Phragmatobia*, *Phaos*, *Metacrias* and the South African species recently placed in *Pseudophragmatobia* Krüger (Krüger 2009).

Here, we review the Andean species of *Phragmatobia*, and place most of these species in two new genera, *Andesobia* gen. n., and *Patagobia* gen. n. The life history of *Andesobia jelskii* (Oberthür, 1881), comb. n.,is described, which shows several remarkable traits presumably in response to the high elevation environment they inhabit. *Phragmatobia karsholti* Toulgoët, 1991 is an unrelated species that is transferred to *Venedictoffia* Toulgoët, comb. n. *Venedictoffia* is neither in the Arctiina nor Spilosomina, and is provisionally transferred to the Phaegopterina.

## Methods and materials

Adult genitalia were prepared following the methods of Lafontaine (2004). Cleaned, stained genitalia were stored and examined in 30% ethanol, and slide-mounted in Euparal before being photographed using a Nikon D200 digital camera.

Repository abbreviations are as follows:

**AMNH** American Museum of Natural History, New York

BMNH The Natural History Museum (formerly British Museum [Natural History]), London

CDFM Collection J. De Freina, Munich

CNC Canadian National Collection of Insects, Arachnids and Nematodes, Ottawa

CPG Collection Pape, Grafenau, Germany

CSO Collection Speidel, Olching, Germany

CTN Collection Tannert, Nuernberg, Germany

NHMB Natural History Museum, Berlin

USNM National Museum of Natural History (formerly United States National Museum), Washington, D.C.

ZSM Zoologische Staatssammlung, Munich

ZMUC Zoologisk Museum, Universitets Copenhagen, Copenhagen

Description of the immature stages and life history was based on four successive generations of laboratory rearings in 2009 and 2010 by JJD, obtained from live material from Peru, Junin region, Huicuash E of Tarma, 11°23'S, 75°53'W, 4100 m. All rearings were conducted indoors at ambient temperatures between 10° C and 23° C. Mortality was extremely low up to F3, but F4 larvae had higher mortality rates and females exhibited reduced fertility, both presumably symptoms of inbreeding. To obtain matings, newly emerged females were placed individually in wooden boxes screened at the top to allow air circulation. Copulation was achieved only under sunny conditions, probably because males are active only during warm, sunny periods under natural conditions. Larvae accepted both dandelion foliage (*Taraxacum officinale* L.) and grass (*Poa* sp.), with a preference for the latter.

We used the 658 bp ‘barcode’ region of the first subunit of the cytochrome oxidase (*cox1*) gene (Ratnasingham and Hebert 2007) of *Andesobia jelskii* to compare to *Phragmatobia* species and other New World genera of Spilosomina. DNA was extracted from one leg removed from a dried specimen, sent to the University of Guelph in dry Eppendorf tubes, and processed as part of the “All Leps Barcode of Life Campaign” (www.lepbarcoding.org). DNA extraction, amplification and sequencing protocols for the Barcode of Life (BOLD) initiative are detailed in Hebert et al. (2003). Haplotypes of all *cox1* ‘barcode’ fragments were compared with phylograms constructed using the neighbour-joining method as implemented on the BOLD website.

## Systematics

### Phragmatobia

Stephens, 1828

http://species-id.net/wiki/Phragmatobia

#### Type species.

*Phalaena fuliginosa* Linnaeus, 1758 (by monotypy).

#### Type locality.

[Europe].

*Phragmatobia* includes five species distributed in the Palaearctic and Nearctic regions (including one Holarctic species, *Phragmatobia fuliginosa* (L.)), with the Neotropical species and one Asian species here transferred to other genera. As suggested by Forbes (1960), *Phragmatobia* is probably most closely related to the Nearctic genus *Pyrrharctia* Packard; male genitalic and molecular characters strongly support these two as sister taxa (Schmidt 2007). We examined all *Phragmatobia* species, i.e. *Phragmatobia fuliginosa* (Linnaeus, 1758), *Phragmatobia amurensis* Seitz, 1910, *Phragmatobia placida* (Frivaldszky, 1835), *Phragmatobia lineata* Newman & Donahue, 1966 and *Phragmatobia assimilans* Walker, 1855. Examination of the type material (BMNH) of *Phragmatobia oberthueri* Rothschild, 1910, described from Kuku-Noor, Tibet, revealed that it is a junior synonym of *Lachana alpherakii* (Grum-Grzhimailo, 1891) [Erebidae: Lymantriinae], syn. n., comb. n., a group recently revised by Trofimova (2008). Two other species sometimes placed in *Phragmatobia* in the recent literature have been transferred to other genera, namely *Orontobia coelestina* Püngeler, 1904 (De Freina 1997) and *Epatolmis luctifera* ([Denis & Schiffermüller], 1775) (Kôda 1988). Watson and Goodger (1986) placed eight Neotropical species in *Phragmatobia*, three of which were transferred to other genera by Ferguson (1985). *Phragmatobia modesta* Maassen, 1890 was recently moved to *Amastus* Walker [Arctiini: Phaegopterina] by Vincent and Laguerre (2010), leaving four Neotropical species that are dealt with here.

#### Diagnosis.

*Phragmatobia* is a fairly homogeneous group characterized by the following combination of characters: male antennae simple; wings fully developed in both sexes, forewing transverse lines diffuse or absent; wing colours varying from pinkish red to dark vinaceous red with darkbrown to blackish markings. Microtymbal of metepisternum well developed (*Phragmatobia fuliginosa*) to obsolete (*Phragmatobia assimilans*). Male genitalia with apical process of valve finger-like and ovoid in cross section; clasper spade-like, oriented transverse to longitudinal axis of valve, originating from inner surface of valve and directed mesad (divided into a ventral and costal lobe in *Phragmatobia fuliginosa*); apex of aedeagus with spinose plates; paired, intersegmental coremata present between sternites 7–8. Females with ductus bursae heavily sclerotized, dorso-ventrally flattened, and nearly as wide as width of abdomen; corpus bursae globose, with two signa consisting of small flattened spicules; dorsal pheromone gland paired, each duct with 3–4 branches, the apices of which are rounded.

### Andesobia

Schmidt & De Freina gen. n.

http://species-id.net/wiki/Phragmatobia

urn:lsid:zoobank.org:act:BA7ACA8C-9856-4B81-AE99-A344DCED0CBC

http://species-id.net/wiki/Andesobia

**Figures 1–4.**
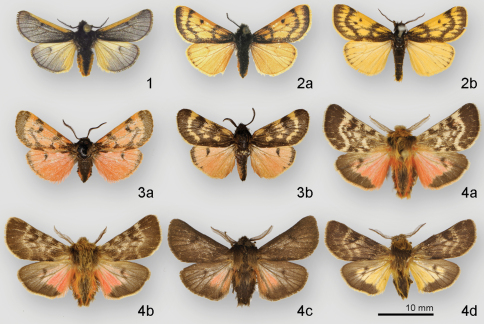
Adult habitus of male *Andesobia* and *Patagobia* species **1** *Andesobia jelskii* **2** *Andesobia boliviana* **2b** *Andesobia boliviana* (holotype of *Estigmene boliviana* Gaede) **3a,b** *Andesobia sanguinea* **4a,b,c,d** *Patagobia thursbyi*.

Figs 1–3 5–10 12 [Fig F5] 18

#### Type species.

*Andesobia jelskii* Oberthür, 1881

#### Etymology.

The name is feminine in gender, formed by combining the words Andes and –*obia* from the generic name *Phragmatobia*.

#### Diagnosis.

*Andesobia* is related to *Patagobia*, but is distinguished by the following combination of characters: eyes reduced and ellipsoid, 1.4–1.6 × as high as wide, gena with broader unscaled area laterally; posterior antennal rami 1.2–1.5 × and anterior rami 1.1–1.5 × longer than segment length (longest anterior and posterior rami 3 × as long as segment in *Patagobia*); 2^nd^ labial segment short and stout, 1.1 × as long as wide, 2 × longer than apical segment; thoracic collar concolourous with dorsal thoracic vestiture (contrastingly paler ochre in *Patagobia*); thoracic vestiture sparse and shaggy, compared to dense and pilose vestiture in *Patagobia*;femur and tibia very stout, 3.0–3.5 × longer than wide compared to 4.5–5.6 × in *Patagobia*; metatibia of *Andesobia* with one pair of spurs, two pairs in *Patagobia*; medial line of forewing absent in *Andesobia*, present in *Patagobia*; postmedial line never double in *Andesobia*, often double in *Patagobia*; hindwing discal spot small and sharpor absent in *Andesobia*, diffuse and more elongate in *Patagobia*. *Andesobia* is endemic to the Puna grasslands of the high Andes of Peru and Bolivia.

#### Description.

Male. ***Head*** – vestiture dark brown to black, shaggy appearance, setae long; antenna weakly bipectinate, ciliate ventrally; longest posterior rami 1.3–2.0 × segment length, longest anterior rami 1.1–1.8 × segment length; rami longest over middle third of antenna, decreasing in length toward base and apex; eye elliptical, 1.4–1.6 × as high as wide; labial palps short, not extending beyond vestiture of frons; 2^nd^ labial segment short and stout, 1.1 × as long as wide, 2 × longer than apical segment; haustellum reduced and poorly sclerotized, presumably non-functional. ***Thorax*** – vestiture of vertex and ventrum of thorax black brown; tegulae and patagia black brown; legs black brown, dorsum of femur ochre or dull pinkish red, co-varying with hindwing and abdomen ground colour; apex of prothoracic tibia with two subequal, blunt, triangular projections; two meso- and metathoracic tibial spurs, posterior spur slightly longer than anterior, length of spurs approximately equal to tibial width at apex; metepisternum with rounded ridge along anterior margin, metepisternal microtymbals absent. ***Forewing*** – relatively small for an arctiine, forewing length 8–13 mm, elongate with apex less rounded than in *Paracles* and *Spilosoma*, length:width ratio averaging 2.2; ground colour ochre yellow, whitish to pinkish red or brownish grey; markings varying from obsoloete (*Andesobia jelskii*) to well defined, grey-brown transverse bands; when present, darker pattern consisting of dark-brown basal area, sub-basal band, discal spot, postmedial band and marginal band; bands occasionally confluent along anal margin; ventrally with bands obsolete except for marginal band, and with a brighter yellowish or reddish ground colour. ***Hindwing*** – ground colour slightly richer yellowish or reddish than forewing, with dark-brown to grey-brown marginal band, varying from nearly obsolete (reduced to intermittent diffuse spots extending from apex halfway to anal angle), to broad and diffuse over distal third of wing; brownish, crescentic discal spot small but usually well defined, sometimes absent; ventrally with dark markings less saturated. ***Abdomen*** – Segments A1–A3 entirely brownish black, remaining segments ochre or reddish subdorsally, with brownish-black dorsal line, widest in *Andesobia flavata*; ventrally, varying from entirely brownish black (*Andesobia sanguinea*) to black with narrow ochre border on distal margin of sternites (*Andesobia flavata*) or entirely ochre (*Andesobia jelskii*); coremata highly reduced to paired patches of sparse, deciduous setae. ***Genitalia*** – highly simplified overall with massive, triangular dorsoventrally flattened uncus characteristic of subtribe; uncus as long as width of base, broadly joined to wide, band-like tegumen; dorsal margin of tegumen caudally recurved; valve simple and digitate, lacking processes or claspers, 1–1.7 × as long as uncus-tegumen complex; vinculum semicircular, saccus v-shaped, similar in length to uncus; juxta evenly convex and hemispherical, dorsal margin slightly narrowed; aedeagus relatively large and stout, 3 × longer than wide, 1.5 × as long as width of genital capsule, curving dorsad 25–30°, proximal end approximately ⅓ narrower than apex; coecum 1/10 length of aedeagus, directed slightly ventrad; vesica directed dorso-distad, globose, finely spiculate, with small basal and poorly differentiated apical diverticulum. **Female** (*Andesobia jelskii* and *Andesobia sanguinea* only; female of *Andesobia boliviana* and *Andesobia flavata* unknown). ***Head*** – antennae 0.5 × as long as that of male, finely biserrate; proboscis atrophied; vestiture of closely appressed, ochre scales, lacking long, shaggy black scales present in males. ***Thorax*** – vestiture similar to that of head, notably lacking ‘shaggy’ appearance of males; legs reduced, 2/3 as long as those of male. ***Forewing and hindwing*** – micropterous and highly reduced, forewing 1.5–2.5 mm long, fully scaled and concoulours with dull tan colour of thorax, but without any discernible wing pattern.***Abdomen*** – light ochre gray with fine, short velvety hairs, tergites well sclerotized, black, giving dorsum of abdomen appearance of a broad, black medial band; ventrally with narrower, lighter grayish-black medial band; integument broad and membranous laterally, allowing for distension caused by ova. ***Genitalia*** (based on *Andesobia jelskii*) – ostium and lamella antevaginalis membranous and poorly defined; lamella postvaginalis consisting of a broad, shallow sclerotized pouch; ductus bursae lightly sclerotized, dorsoventrally flattened, 2 × as long as wide; corpus bursae pear shaped, and relatively small, 2 × as longh as ductus bursae; diameter of distal, globose chamber 2 × width of ductus bursae; signum lacking; ductus seminalis wide and rugose, bulla seminalis large, diameter 1.5 × that of corpus bursae; posterior apophysis equal in length to papillae anales, anterior apophysis 0.6 × as long as papillae anales; each paired dorsal pheromone gland consisting of two tree-like subdivisions, each subdivision with 3–5 smaller diverticula.

**Figures 5–6.**
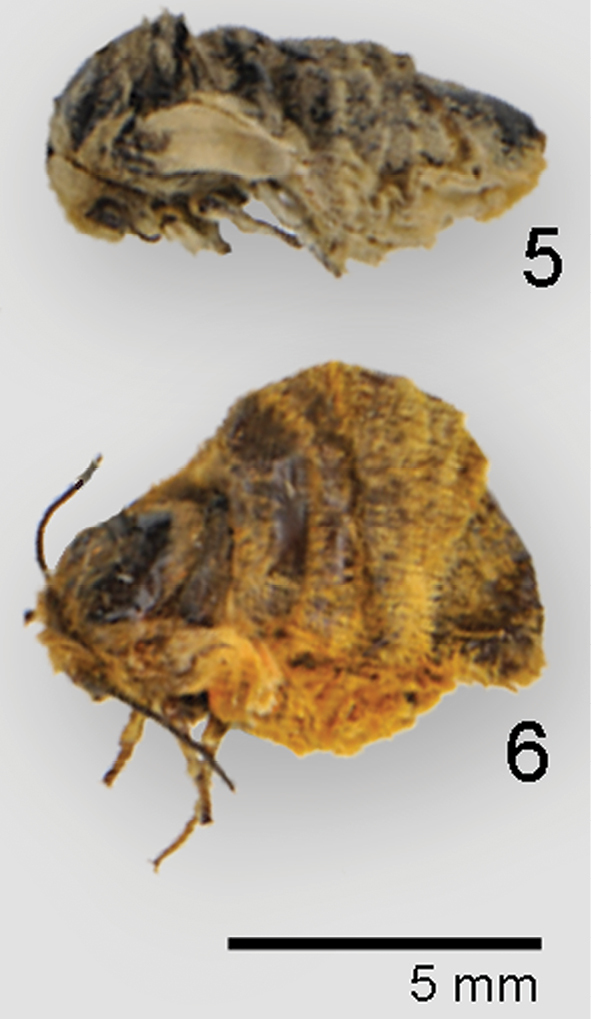
Adult habitus of female **5** *Andesobia jelskii* **6** *Andesobia sanguinea*.

#### Remarks.

Structurally, *Andesobia* is quite homogeneous, the main species-level differences occuring in the length and shape of the male valve and the vesica. The highly simplified, digitate male valve and massive uncus-tegumen compex is shared with several other Neotropical genera including *Paracles*, *Patagobia*, *Caribarctia* Ferguson and *Leichosila* Schmidt. The mtDNA barcode sequence (*Andesobia jelskii*) does not provide any additional resolution of relationships within this group, with minimum pairwise distances (uncorrected) between *Andesobia*, *Paracles*, *Phragmatobia*, *Leichosila*, *Caribarctia* and *Phaos* ranged from 6–8%. Sequences for *Patagobia* were not available.

Several Andean species are superficially similar to *Andesobia* and *Patagobia*, and require comment. *Paracles herbuloti* (Toulgoët), *Paracles minuta* Becker & Miller, and *Paracles diminuta* Becker & Miller are small species with a simple or highly reduced forewing pattern. Females of all three are unknown, but the structurally similar and probably congeneric *Chilesia anguloi* Ruiz, *Chilesia rudis* (Butler) and *Chilesia watsoni* Ruiz have micropterous females (Ruiz 1989; Vargas and Parra 2003). Despite these similarities to *Andesobia* (and *Patagobia*), the broader, more rounded wings, shorter, rounder saccus, greatly elongated tegumen, very short valva, and small vesica are consistent with those of other *Paracles* species, and not with *Andesobia* or *Patagobia*.

#### Biology and distribution.

Data on the biology of *Andesobia* is based primarily on *Andesobia jelskii* and is discussed in more detail under the species account below. *Andesobia* is adapted to cold-temperate alpine habitats, males flying during sunny periods and the females being micropterous. Mating and oviposition occurs inside the female cocoon. The female-biased sex ratio of the broods reared during this study may indicate that females are capable of parthenogensis, as in some other cold-adapted flightless Lepidoptera (Suomalainen 1962). Adults emerge during the middle of the four-month wet season in the otherwise xeric grassland habitat. *Andesobia* is endemic to the Puna grasslands of the high Andes, occuring from central Peru south to the Lake Titicaca region of southern Peru/Bolivia.

### Andesobia jelskii

(Oberthür, 1881) comb. n.

http://species-id.net/wiki/Andesobia_jelskii

Figs 1 5 12–16 18

Arctia jelskii Oberthür 1881: 33, pl. X, f. 3. Male holotype [BMNH]. Type locality: [Peru], Junin.
Mallocephala imitatrix Rothschild 1922: 493, comb. n, syn. n. 16 male and 5 female syntypes [BMNH]. Type locality: Peru, Junin.
Mallocephala imitatrix ab. *griseola*Rothschild 1922: 493, comb. n, syn. n. Holotype male [BMNH]. Type locality: Peru, Junin.
Mallocephala imitatrix ab. *luteola*Rothschild 1922: 493, comb. n, syn. n. 7 female syntypes [BMNH]. Type locality: Peru, Junin.

#### Material examined.

We examined over 150 specimens, obtained through four successive lab-reared generations originating from Peru, Junin region, Huicuash E of Tarma, 11°23'S, 75°53'W, 4100 m. Vouchers are deposited in CDFM, ZSM, CNC, CPG, CTN. Two specimens were included for DNA barcode analysis, voucher numbers CNC LEP 68032 (no GenBank accession number available) and CNC LEP 68033 (GenBank accession # HM416594) [CNC].

#### Diagnosis and re-description.

*Andesobia jelskii* was omitted from the catalogue of Neotropical Arctiinae (Watson and Goodger 1982), probably because Hampson (1901) placed it in the Palaearctic genus *Ocnogyna*. This species is most similar to *Andesobia flavata* and *Andesobia boliviana*, but lacks any trace of transverse forewing lines and has an uninterrupted ochre costal band, which is interrupted by the antemedial and postmedial lines in other *Andesobia* species; the hindwing marginal band has a diffuse inner border and is much wider than in other species of *Andesobia*, extending at least to the discal area. Internally, the male valve is shorter and wider, appropximately 6 × longer than the narrowest diameter compared to 6.7–8.0 × longer in *Andesobia flavata*.

A detailed morphological description is given in the generic account of *Andesobia*, and the following description addresses characters specific to *Andesobia jelskii*. **Male**. ***Head*** – antenna (Fig. 9a) with posterior rami 1.6–1.9 × segment length, longest anterior rami 1.4–1.8 × segment length; eye elliptical, 1.4–1.6 × as high as wide. ***Thorax*** –vestiture and legs black brown, dorsum of femur ochre. ***Forewing*** – forewing length average 11 mm, range 8–12 mm; ground colour brownish grey with yellowish-ochre costal band varying to entirely dark brownish grey or plae ochre grey (type of *luteola*), indistinct black discal spot, other markings obsolete ventrally with paler yellowish ochre ground colour. ***Hindwing*** – ground colour yellowish ochre with broad, diffusely bordered grey-brown marginal band over distal third of wing, varying to entirely dark brownish grey; brownish, crescentic discal spot small but well defined; ventrally with dark markings less saturated. ***Abdomen*** – segments A1–A3 brownish black, remaining segments ochre subdorsally, with brownish-black dorsal line; ventrally entirely ochre. ***Genitalia*** (Figs 9b,c) – valve digitate, slightly flattened laterally and narrowing slightly medially; equal in length to uncus-tegumen complex; vinculum semicircular, saccus v-shaped, similar in length to uncus; aedeagus relatively large and stout, 3 × longer than wide, 1.5 × as long as width of genital capsule, curving dorsad 25–30°, proximal end approximately ⅓ narrower than apex; coecum 1/6–1/8 length of aedeagus, directed slightly ventrad; vesica directed dorso-distad, globose, finely spiculate, with poorly differentiated apical diverticulum. **Female** (Figs 5, 10, 13, 16). Described above in the genus account for *Andesobia*; differing externally from *Andesobia sanguinea* by the lack of yellowish-orange flush present in *Andesobia sanguinea*, particuarly on the ventral and lateral surfaces of the abdomen.

#### Immature stages.

*Egg* – almost spherical, poles only very weakly flattened; micropyle very weakly sculptured, barely visible; ivory white changing to dark greyish white prior to hatching. *Larva* – 1^st^ instar larva initially translucent white, becoming opaque white; setae black, yellow orange prior to molting. 2^nd^ instar integument black, more densely setose than 1^st^ instar. 3^rd^ instar with jet black setae, except rusty brown on A2–A5; spiracles white. 4^th^ instar (Figs 15a, b) with verrucae more pronounced than in previous instar; setae jet black with silver sheen apically, somewhat lighter smoky grey subventrally; colour of setae polymorphic in last instar, either with A2–A5 yellowish orange and segments A6–A8 with subdorsal and lateral silvery-white setae mixed in (Fig. 15a), or with orange setae very dark brown to black (Fig. 15b); when mature, female larva about twice as large as male larva. *Pupa* – cremaster short, penicillate, head compact with short, stiff setae (Fig. 12). Cocoon spherical to ovoid, reddish brown to dull brown, consisting of a single, thin and flimsy layer with incorporated larval setae (Fig. 16).

**Figures 7–11.**
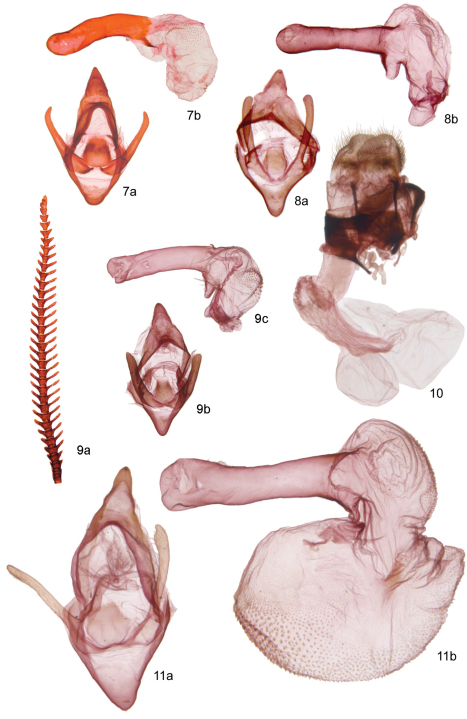
Genitalic and antennal morphology of *Andesobia* and *Patagobia*. **7** *Andesobia sanguinea* **8** *Andesobia boliviana* **9** *Andesobia jelskii* (male) **10** *Andesobia jelskii* (female) **11** *Patagobia thursbyi*.

**Figures 12–16.**
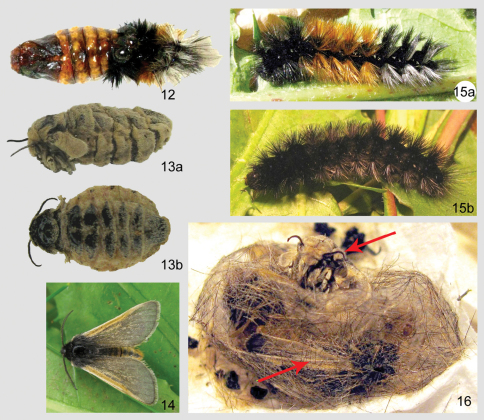
*Andesobia jelskii*.**12** female pupa **13a** female, lateral aspect **13b** female, dorsal aspect **14** male **15a** mature larva **15b** mature larva, dark form **16** male (lower arrow) and female (upper arrow) *in copulo* inside coccoon of female.

#### Biology and distribution.

**Figure 17. F5:**
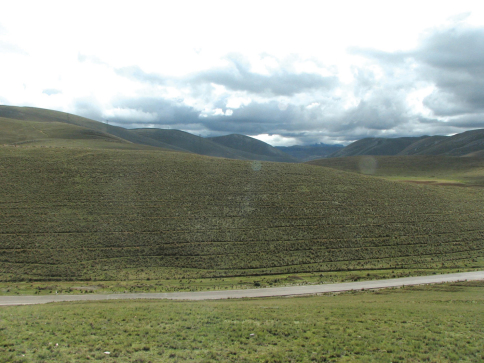
Habitat of *Andesobia jelskii* near the type locality, Junin region, Peru (photo J. Klir).

**Figure 18.**
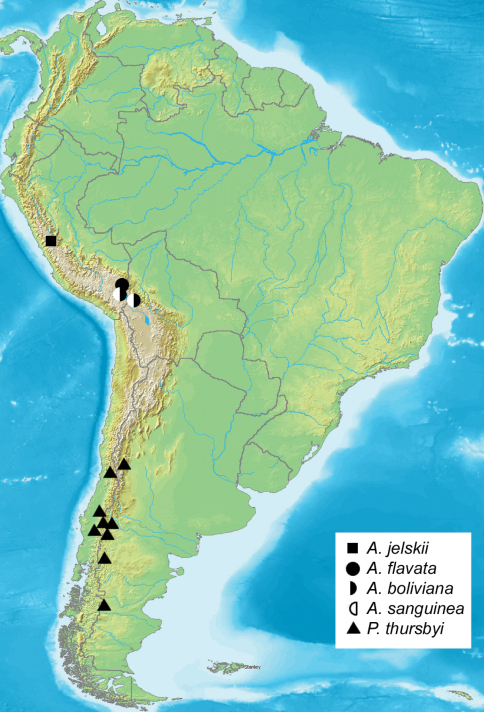
Distribution of *Andesobia* and *Patagobia*.

Eggs whitish, turning dark grey three days prior to hatching, hatching in about 10 days. First instar larvae initially feed on the tissue of the dead or dying female, then leave the cocoon in search of plant material. Duration of the first instar is six days, second instar five to six days. *Taraxacum* F.H. Wigg. and lawn grass (*Poa* L.) are both acceptible food plants in captivity, suggesting that larvae are polyphagous in nature. Notably, larvae emit an unpleasant odour of decay when disturbed. In late instars, female larvae are twice as large as male larvae. During the first three instars, larvae avoided sunlight, but the last two instars showed increased tolerance to sunlight, possibly to accelerate development. Cannibalism was not observed even at higher densities. Males pupated sooner than females, but the pupal stage is shorter in females lasting only a few days, so emergence of both sexes is more or less synchronous. Cocoons (Fig. 16) were spun between leaves of the food plants near the ground. The moths emerge in the morning, with relatively fast expansion of the wings. Females remain in the loosely-spun cocoon, and presumably emit mating pheromones from within the thin cocoon soon after emerging from the pupa, because males dig through the loose silk webbing to enter and mate inside the cocoon (Fig. 16). The pair remains in copula for several hours, after which the male leaves the cocoon, and the female deposits about 50 eggs inside the cocoon. Males are diurnal and fly rapidly during sunny periods. Reared cohorts of *Andesobia jelskii* displayed an unequal sex ratio of about 5 female: 3 male; female microptery and a female-biased sex ratio is associated with parthenogenesis in other families (Heterogynidae, Psychidae, Lymantriinae: *Teia* Hübner), and *Andesobia* may also be capable of parthenogenesis, which has not been documented in the Arctiinae. *Andesobia jelskii* is currently known only from the Junin region of Peru (Fig. 17), at 4100 m elevation in the Puna grassland ecoregion of the central Andes (Fig. 18). Like other members of the genus, the flight period is early in the year (January), in the middle of the four-month wet season.

### Andesobia flavata

(Hampson, 1901) comb. n.

http://species-id.net/wiki/Andesobia_flavata

Maenas flavata Hampson, 1901: 512, pl. 51, f. 7. Male holotype [BMNH]. Type locality: Peru, Limbane [Limbani], 6000 ft.

#### Diagnosis.

Very few specimens of *Andesobia flavata* are known, and it is closely related to or conspecific with *Andesobia boliviana*. Externally, the holotype of *Andesobia flavata* differs from *Andesobia boliviana* only in having a slightly broader and more diffuse forewing marginal band, suggestive of minor intraspecific variation. However, the genitalic structure of the holotype (BMNH genitalia slide # ARCT:3421) reveals slight differences compared to *Andesobia flavata*, namely a slightly shorter, wider valve and a lack of the fine spicules present on the vesica of *Andesobia flavata*. Additional study material is needed to properly evaluate the status of these two taxa.

### Andesobia boliviana

(Gaede, 1923) comb. n.

http://species-id.net/wiki/Andesobia_boliviana

Figs 2 8 18

Estigmene boliviana Gaede 1923: 20. Male holotype [NHMB] (Fig. 2b). Type locality: Bolivia, La Paz.
Turuptiana flavescens Rothschild 1933: 188, syn. n. Male lectotype, here designated [BMNH]. Type locality: [Peru / Bolivia], Lake Titicaca.

#### Type material.

The three examined male syntypes of *Turuptiana flavescens* exhibit variation in the extent of the forewing markings, two specimens closely approaching the appearance of the *Andesobia flavata* holotype (see also Remarks under *Andesobia flavata*). The third syntype labeled “type” with a round, red-bordered label and a blue label reading “genitalia slide no. 3422” is here designated as lectotype; it is an almost exact match to the two specimens illustrated here (Fig. 2).

#### Remarks.

Subsequent to its description, *Estigmene boliviana* (Fig. 2b) disappeared from the literature. Although appearing in the print version of the Zoological Record for 1923, it is absent from the digital version of Zoological Record and the card index of the BMNH (Global Lepidoptera Names Index 2011). It was also omitted by Watson and Goodger (1986). The holotype label data is as follows: “La Paz / Bolivia / 95 Garlepp”; “als Puppe / Nov. 8. larva”; “1855a”; “Coll. / Staudinger”; “Estigmene / boliviana G. / E. Rschau [Entomologische Rundschau] B40 S20.”; “Type”.

### Andesobia sanguinea

(Hampson, 1907) comb. n.

http://species-id.net/wiki/Andesobia_sanguinea

Figs 3 6 7 18

Turuptiana sanguinea Hampson, 1907: 238. Type locality: Bolivia, La Paz, 9000’. Male lectotype, here designated [BMNH].

#### Type material.

Hampson (1907) based his description on what he believed to be a male and a female, but both specimens (BMNH) are males. One syntype is in rather poor condition with rubbed, partially broken wings, lacking antennae and abdomen. The second syntype, in excellent condition, labeled “Type” with a round, red-bordered label and a blue label reading “genitalia slide no. 3423” is here designated as lectotype to ensure the stability of the name.

#### Diagnosis.

*Andesobia sanguinea* is the only member of the genus with red colouration, prevalent on the hindwing and often the forewing, the latter varying from whitish pink (Fig. 3a) to whitish tan (Fig. 3b). Females are micropterous and are similar to *Andesobia jelskii*, but with a more yellowish colour (Fig. 6). The biology is unknown. It appears to be sympatric with *Andesobia boliviana*, and is known only from the Lake Titicaca region (Fig. 18).

### Patagobia

Schmidt & De Freina gen. n.

urn:lsid:zoobank.org:act:AE8678AA-E7C1-4A86-8C25-7E539E5998DB

http://species-id.net/wiki/Patagobia

#### Type species.

*Turuptiana thursbyi* Rothschild, 1910.

#### Etymology.

The name is derived from a combination of the words Patagonia and *Phragmatobia*.

#### Diagnosis.

Although *Patagobia* shows similarities to the Holarctic *Phragmatobia* in some external aspects, it differs in having longer, symmetrical rami of the male antenna, ochre thoracic collar, lack of a male clasper, pale tan forewing pattern (usually), and a restricted distribution to the Chilean Andes of South America. The wing colour and pattern is also similar to *Andesobia*, but structurally *Patagobia* has a more robust build with denser thoracic vestiture, equally long posterior and anterior male antennal rami (anterior rami shorter than posterior in *Andesobia*), male antennal rami up to 3 × longer than antennal segment length (up to 2 × in *Andesobia*); 2^nd^ labial segment elongate, 1.8 × as long as wide, 1.5 × longer than apical segment; thoracic collar contrastingly paler ochre (conconcolourous with dorsal thoracic vestiture in *Andesobia*); thoracic vestiture dense and pilose (sparse and shaggy in *Andesobia*);femur and tibia elongate, 4.5–5.6 × longer than wide (very stout, 3.0–3.5 × longer than wide in *Andesobia*); metatibia with two pairs of spurs (one pair in *Andesobia*); forewing medial line present (absent in *Andesobia*); postmedial line usually double (absent in *Andesobia*); hindwing discal spot diffuse and elongate (sharp or absent in *Andesobia*). The male coremata betwen the 7^th^ and 8^th^ sternite are moderately developed in *Patagobia*, very reduced in *Andesobia*.

#### Description.

Male (female unknown). ***Head*** – vestiture dark brown to black, setae long; antenna bipectinate, ciliate ventrally; longest posterior rami 1.5–3.0 × segment length, longest anterior rami 1.1–3.0 × segment length; rami longest over middle third of antenna, decreasing in length toward base and apex; eye elliptical, 1.2–1.5 × as high as wide; labial palp short, not extending beyond vestiture of frons; haustellum reduced and poorly sclerotized, presumably nonfunctional. ***Thorax*** – vestiture of vertex and ventrum of thorax black brown; tegulae entirely black brown or black brown edged with yellowish brown; patagia yellowish brown or rarely black brown; leg vestiture brownish ochre, dorsum of femur yellow or red, co-varying with hindwing and abdomen ground colour; apex of prothoracic tibia with two subequal, blunt, triangular projections; mesotibia with two apical and two subapical spurs, length of apical spurs 1.5 × and supapical spurs 0.6 × tibial width at apex; two metatibial spurs, posterior spur slightly longer than anterior; metepisternum lacking microtymbals. ***Forewings*** – forewing length 12.9–13.2 mm (mean 13.1 mm; *n* = 4), length:width ratio averaging 2.1; ground colour pale ochre yellow but with broad, sometimes entirely confluent dark-brown transverse bands (Fig. 4c); pattern elements consisting of dark basal area and sinuous, diffuse dark-brown transverse lines (Fig. 4a) discal spot indistinct dorsally, but well defined ventrally; ventrally with bands obsolete except for marginal band. ***Hindwing*** – ground colour pinkish red or rarely yellow (Fig. 4d), with broad dark-brown marginal and costal band; well-defined brown, crescentic discal spot; similar ventrally but with discal spot better defined. ***Abdomen*** – Vestiture brownish black and pinkish red or yellow subdorsdally, ventrally with segmental margins yellowish ochre; abdomen entirely dark brown in melanic specimens (Fig. 4c). Coremata between sternites 7–8 in shallow pockets, scent scales approximately 0.5 × as long as sternite length. ***Genitalia*** – highly simplified, with large, triangular, dorsoventrally flattened uncus characteristic of subtribe; uncus 1.5 × longer than width of base, broadly joined to wide, band-like tegumen; dorsal margin of tegumen caudally recurved; valve simple and digitate, lacking processes or claspers, 1.5 × as long as uncus-tegumen complex; vinculum semicircular, saccus v-shaped, similar in length to uncus; aedeagus large, 5–6 × longer than wide, 2 × as long as width of genital capsule, curving dorsad slightly; coecum 1/10 length of aedeagus, directed slightly ventrad; vesica extremely large, diameter when inflated 2 × that of genital capsule; vesica directed right-laterad, globose, finely spiculate, with poorly differentiated basal chamber and large apical chamber (Fig. 11b).

### Patagobia thursbyi

(Rothschild) comb. n.

http://species-id.net/wiki/Patagobia_thursbyi

Turuptiana thursbyi Rothschild 1910: 176, comb. n. Five male syntypes (BMNH). Type locality: [Argentina], Patagonia, Chubut, Valley de Lago Blanco.
Phragmatobia thursbyi pluto Toulgoët, 1987: 241, syn. n.

#### Type material.

Male holotype (ZMUC). Type locality: Argentina, Rio Negro, San Carlos de Bariloche, Colonia Suiza, 810 m.

#### Diagnosis.

The taxon *pluto* Toulgoët has been treated as a subspecies distinct from nominate *thursbyi* based on the nearly unicolourous forewing, resulting from the confluence of the transverse bands. Genitalic structure of both taxa is identical (Toulgoët 1987). Examination of series of specimens from a single locality (Fig. 4b-d) shows that there is considerable variation in the extent of forewing banding, and also in the hindwing ground colour. We therefore treat *pluto* as a synonym of nominate *Patagobia thursbyi*.

#### Remarks.

No detailed habitat information is available for *Patagobia thursbyi*, but locality information shows that it occurs from about 800 m elevation at the southern range edge (46°S) to 2700 m farther north (33°S), corresponding to temperate montane woodlands and grasslands of Patagonia. This region is well known for its high level of endemic species, and circumantarctic tree genera such as *Araucaria* Juss. and *Nothofagus* Blume (World Wildlife Fund 2001). Examined specimens and literature records (Fig. 18) are as follows **Chile -** Malleco Prov.: Cordillera las Raices, Lonquimay, 1050 m (CNC, ZSM); La Fusta, 1200 m (CNC, ZSM); Cordillera Lonquimay, Icalama 1000 m (AMNH, ZSM); Termas de Rio Blanco (Ruiz 1989, ZSM); Cautin region (Ruiz 1989); Nuble Prov.: Chillan, (Ruiz 1989); Santiago: Cantillana [highlands], [Laguna de] Aculeo (Toulgoët 1987). **Argentina** - Chubut Prov.: Valle del Lago Blanco (BMNH, ZSM); Neuquen Prov.: Pampa Tromen, Huayilon (Ruiz 1989, ZSM); San Martin de los Andes (ZSM); Aluminé, 1200 m (Toulgoët 1987); Rio Negro Prov.: San Carlos de Bariloche, Colonia Suisa, 810 m (Toulgoët 1987, ZSM); Paso Flores (ZSM); Mendoza region, 2750 m (Toulgoët 1987).

### Venedictoffia karsholti

(Toulgoët, 1991) comb. n.

http://species-id.net/wiki/Venedictoffia_karsholti

Phragmatobia karsholti Toulgoët, 1991: 18. Holotype male (ZMUC). Type locality: Peru, Ancash 35 km SE de Huaraz, Cerro Cahuish, 4100 m, Quebrada Pucavado.

#### Diagnosis.

Toulgoët (1991) described this species in *Phragmatobia* without further elaboration of this generic placement. The wing shape, forewing pattern, and male genitalic structure (pedunculate uncus, bipartite valve) of *karsholti* is shared with *Venedictoffia* Toulgoët (Toulgoët 1977), so *karsholti* is transferred to *Venedictoffia* (comb. n.). Although the simple forewing pattern, reduced size, and bipectinate male antenna show some resemblance to *Andesobia* and *Patagobia*, male genitalic structure (see Toulgoët 1991) shows that *Venedictoffia* does not belong in the subtribe Spilosomina nor in the Arctiina. The caudally recurved dorsal margin of the tegumen and lateral lobes of the 8th sternite, autapomorphies of the Spilosomina (Schmidt 2007), are lacking.The unusual pedunculate uncus, scoop-shaped tegumen and bipartite valve are traits exhibited in numerous Neotropical Phaegopterina and some Pericopina. *Venedictoffia* is therefore provisionally transferred to the Phaegopterina.

## Discussion

In his global review of Arctiina genera (as Arctiini), Ferguson (1985) divided the genera among five groups, namely “*Neoarctia*-*Grammia*”, “*Holomelina*” (now *Virbia* Walker; Zaspel and Weller 2006), “*Arctia*-*Hyphoraia*”, “*Spilosoma*” and “*Phragmatobia*-*Ocnogyna*”. The latter two do not form natural groups, but are monophyletic as a whole and were treated as subtribe Spilsomina by Schmidt (2007), with the exclusion of *Kodiosoma* Stretch, which is related to the *Euchaetes* group (Schmidt 2007; Schmidt and Opler 2008). Ferguson considered the Andean “*Phragmatobia*” species to be closely related to and possibly congeneric with *Metacrias* Meyrick, 1887 of New Zealand. *Metacrias* is probably congeneric with *Phaos* Walker, 1855 of Australia / Tasmania (Patrick et al. 2003). *Patagobia* is structurally more similar to *Metacrias*/*Phaos* than to *Andesobia*, the latter apparently representing a more ancient split from *Patagobia* + *Metacrias* / *Phaos* (PMP) lineage. This implies that the Andean group is not monophyletic, and that the PMP group has a circumantarctic distribution. The biology of the PMP group provides additional evidence for a common ancestry; all inhabit mesic, cool-temperate habitats, particularly grassland and tussock tundra, with specialized adaptations such as diurnal flight (males) and flightless females. Land connections between southern South America and Australia existed as late as the Late Eocene (~35 MYA) (McLoughlin 2001), but the biogeography of New Zealand *Metacrias* is more difficult to explain, since Zealandia separated from other Gondwanan landmasses about 80 MYA (McLoughlin 2001). Transoceanic dispersal events would be highly unlikely for the flightless females, and early instar larvae are not known to exhibit ballooning behaviour (wind dispersal by silk strands) (Gibbs 1962). The zoogeography of this fascinating group awaits further study.

## Supplementary Material

XML Treatment for Phragmatobia

XML Treatment for Andesobia

XML Treatment for Andesobia jelskii

XML Treatment for Andesobia flavata

XML Treatment for Andesobia boliviana

XML Treatment for Andesobia sanguinea

XML Treatment for Patagobia

XML Treatment for Patagobia thursbyi

XML Treatment for Venedictoffia karsholti
